# Is Panenteric Pillcam^TM^ Crohn’s Capsule Endoscopy Ready for Widespread Use? A Narrative Review

**DOI:** 10.3390/diagnostics13122032

**Published:** 2023-06-12

**Authors:** Alexandros Toskas, Faidon-Marios Laskaratos, Sergio Coda, Saswata Banerjee, Owen Epstein

**Affiliations:** 1 St Mark’s Hospital, Watford Rd., Harrow HA1 3UJ, UK; 2 Digestive Diseases Centre, Queen’s Hospital, Barking Havering and Redbridge University Hospitals NHS Trust, Rom Valley Way, Romford RM7 0AG, UK; flaskaratos@nhs.net (F.-M.L.); sergio.coda@nhs.net (S.C.); saswata.banerjee@nhs.net (S.B.); 3 Royal Free Hospital, Pond St., London NW3 2QG, UK; o.epstein@btinternet.com

**Keywords:** inflammatory bowel disease (IBD), PillCam, Crohn’s capsule endoscopy, panenteric imaging

## Abstract

Patients diagnosed with Crohn’s disease are increasingly subjected to repeat colonoscopic and radiological examinations to assess the extent of the disease severity and the effects of treatment. Pillcam^TM^ Crohn’s video capsule, a modified colon capsule, was developed to generate a minimally invasive mouth to rectum video of the gastrointestinal tract. The capsule provides a wide-angle panoramic mucosal view to assess inflammation, ulceration, stenosis, disease extent, and effect of treatment. This review summarizes the evidence of its utility in both adult and paediatric Crohn’s disease and reviews the scoring systems used to quantify findings. The literature survey indicates that the Pillcam^TM^ Crohn’s capsule offers high sensitivity and specificity for the detection of inflammatory lesions and the extent and distribution of disease, and it could be considered a reliable imaging modality in both adults and childhood with Crohn’s disease.

## 1. Introduction

Capsule endoscopy is a minimally invasive wireless device capable of acquiring high quality video of the digestive tract mucosa. Single-camera small bowel video capsule endoscopy (SBCE) was introduced in 2001, enabling examination of the small bowel mucosa. This device allowed for minimally invasive examination of the small intestine in patients presenting with obscure gastrointestinal bleeding and for evaluating the extent and severity of small bowel Crohn’s disease (CD). Two subsequent generations of SBCE were developed to improve battery efficiency, field of vision, and image resolution. A number of publications have reported the superiority and safety of SBCE for the grading of the severity and extent of small bowel CD, and the third-generation capsule is now firmly established in the Crohn’s disease management pathway [1,2,3,4,5,6,7,8,9].

Incremental innovation has seen the development of an oesophageal, upper gastrointestinal, and colon capsule, each designed to maximise imaging of the oesophagus, stomach, and colon respectively. Recognising the potential for a single-study panenteric capsule to survey the mucosal surface of both the small and large intestines in CD, Pillcam^TM^ Crohn’s capsule (PCC) (Medtronic, Dublin, Ireland) was developed as a modification of PillCam Colon 2 and was released in 2017. PCC combines forwards and rear-facing wide-angle cameras providing a 344°-wide mucosal view. With the elimination of the Colon Capsule’s sleep mode and by employing an intelligent adaptive frame rate function, efficient power management has maximised the video acquisition time, which, together with a booster regimen to accelerate transit time, is sufficient in most patients to image the mucosa from mouth to rectum [6,10].

The aim of this review is to summarise published reports on the utility of PCC in adults and children with CD. Scoring metrics are considered, and the review offers a backdrop for the current status of PPC in guiding management [8,9,11].

## 2. Materials and Methods

**Figure 1:**
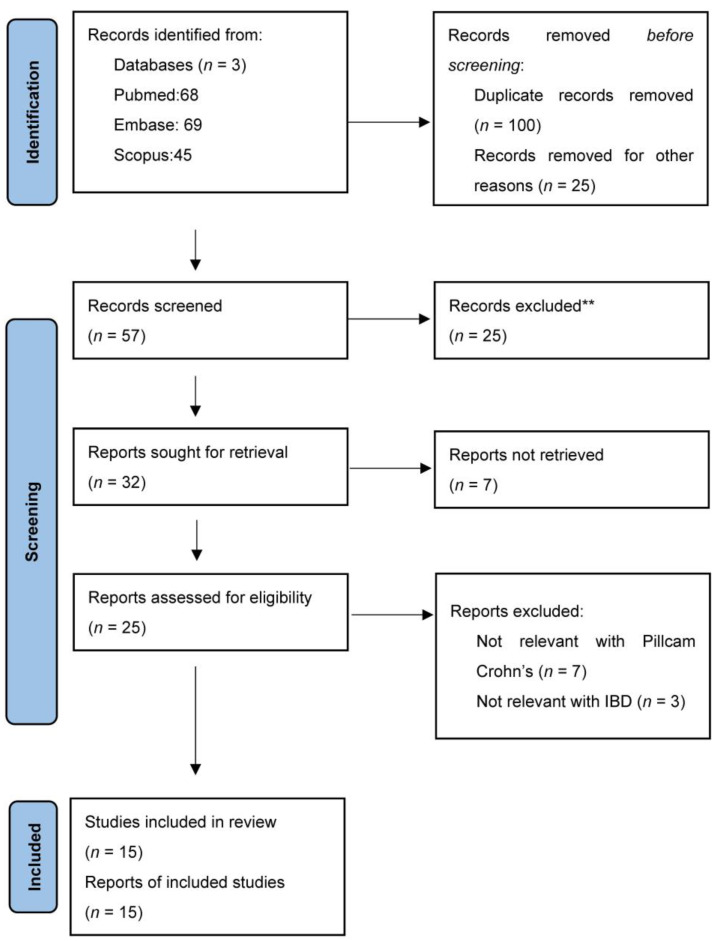
**PRISMA** flow diagram illustrating methodology of literature search. ** Not relevant to panenteric endoscopy and focusing on large bowel findings other than IBD.

A literature search was conducted using Pubmed, Scopus, and Embase using the keywords ‘Panenteric’, ‘Capsule’, and ‘Crohn’s disease’; and 57 results were identified and analysed using the PRISMA flowchart. Twenty-five reports were excluded after abstract review since they were not relevant to panenteric capsule endoscopy, used older capsule versions, or focused on large bowel findings other than CD. With further screening, 15 reports were considered suitable for evaluation and are included in this review (Figure 1). In addition, bowel preparation prokinetic and small bowel booster regimens were recorded, and PCC scoring systems were considered and compared to the Lewis score (LS), which is used extensively for SBCE assessment of small bowel CD.

## 3. Results

### 3.1. Panenteric Capsule Endoscopy in Adults with Inflammatory Bowel Disease

Table 1 provides a summary of the studies investigating the role of PCE in the assessment and management of IBD in adults.

In a recent study by Brodersen et al. only a small proportion of patients (17%) were able to tolerate the entire volume (4 L) of PEG solution. Although the volume of PEG was associated with the obtained image quality, the diagnostic yield was not affected [19]. The bowel preparation and prokinetic and booster regimens used in various studies are summarized in Table 2, indicating a lack of standardisation.

Leighton et al. reported the per-subject diagnostic yield for active CD lesions in 114 patients. They reported 83.3% for PCC and 69.7% for IC (yield difference, 13.6; 95% confidence interval (CI), 2.6–24.7%), while the per-segment diagnostic yield rate was 40.6% for PCC and 32.7% for IC (yield difference 7.9; 95% CI, 3.3–12.4%) [12].

Eliakim et al. evaluated the utility of PCC in patients with established or suspected CD [13]. All 41 videos met the primary endpoint of a successful procedure, that is, whole bowel high quality video imaging suitable for review and report generation. There was no capsule retention. Adler et al. in their study compared the efficacy of PCE versus colonoscopy in evaluating disease activity in UC patients. In 95.7% of cases, there was agreement in Mayo activity index [14].

A large prospective multicentre study by Bruining et al. enrolled 158 subjects from 21 sites and compared the use of the PCC to MR enterography and colonoscopy for the assessment of Crohn’s disease mucosal activity and extent [15]. The study demonstrated that, overall, PCC sensitivity for the detection of enteric inflammation was 94% compared with 100% for MRE and/or IC (*p* = 0.125), but the specificity was significantly higher (74% compared to 22% for MRE and/or IC (*p* = 0.001)). In the segmental analysis, PCC was significantly more sensitive and specific compared to MRE in the proximal small bowel with sensitivity of 97 vs. 71% (*p* = 0.021) and specificity of 87 vs. 66% (*p* = 0.020), respectively. In the terminal ileum (TI), there was no significant difference in sensitivity between PCC and the combined modalities (MRE and/or colonoscopy) or between PCC compared with either MRE or IC alone. The specificity was higher for PCC compared to MRE combined with IC (82 vs. 37%, *p* < 0.001) and MRE alone in the TI. In the colon, there was no significant difference between CE and IC. The study also measured patient satisfaction. Of the 118 subjects who completed the questionnaire, 54% of patients preferred capsule endoscopy, 36% preferred colonoscopy, and 9% preferred the combined modalities of colonoscopy and MRE. The most common reasons for preferring capsule endoscopy were the absence of intravenous access, avoidance of sedation, analgesia and a home escort, the ability to evaluate the entire GI tract in a single procedure, and the ability to remain active during the procedure [15].

In a multicentre, European, observational study, Tai et al. evaluated PCC in the assessment of disease severity and extent in 93 patients with established (*n* = 71) or suspected (*n* = 22) CD. The Montreal classification was upstaged in 33.8% of patients with established Crohn’s disease, and they reported that PCC resulted in management changes in 38.7% (36/93) of patients. Proximal small bowel disease was upstaged in 12.7% and predicted the escalation of therapy. The authors concluded that PCC was feasible in routine practice and allowed for better estimation of severity and extent, which in turn helped to determine the need for treatment escalation [16].

In 2020, Tontini et al. reported the added value of the panoramic 344° view obtained with the use of a forwards and rear facing camera of PCC [8] compared to the standard 172° single-camera view provided by SBCE. PCC completion rates were 90% with an average capsule operating time of 11.8 ± 3.3 h. No safety or technical issues were observed. The panoramic 344° view detected 17.1% more patients with relevant lesions (56.1 vs. 39.0%; *p* = 0.023), resulting in a 16.6% increase in the mean Lewis score (222.8 vs. 185.7%; *p* = 0.031), and in 17.1% of patients, PCC led to an alteration of clinical management (48.8 vs. 31.7%, *p* = 0.023) [8].

Volkers et al. assessed the ability of PCC to measure changes in mucosal disease activity before and after starting biologic treatment. Patients with clinically and biochemically active disease had PCC at baseline and 8–12 weeks after starting their biologic treatment with infliximab, adalimumab, or vedolizumab. PCC remission was observed in 6 of 22 (27%) patients, and 13 of 22 patients (59%) showed a response for both the simple endoscopic score for Crohn’s disease (SES-CD) and the Crohn’s disease endoscopic index of severity (CDEIS). There were also no adverse events. The authors concluded that PCC is useful to assess changes in mucosal disease activity in CD patients [17].

### 3.2. Pantenteric Capsule Endoscopy in Paediatric Inflammatory Bowel Disease

The utility of PCC in paediatric patients with CD was assessed by Oliva et al. [18]. In a study of 48 children, biomarkers, imaging, and PCC evaluations were performed at baseline and after 24 and 52 weeks. The primary endpoint was the ability of PCC to assess mucosal healing and deep remission and to support the treatment options. Based on PCC findings, active inflammation was found in 34 patients (71%) at baseline, 22 patients (46%) at week 24, and 18 patients (39%) at week 52. PCC led to treatment changes in 34 patients (71%) at baseline and 11 patients (23%) at 24 weeks. Using the ‘treat to target’ strategy, proportions of patients with mucosal healing and deep remission increased from 21% at baseline to 54% at week 24 and 58% at week 52. The authors suggested that, in paediatric CD, PCC could play an important role in monitoring mucosal inflammation, guiding therapy, and obtaining higher rates of mucosal healing and deep remission over 52 weeks [18].

### 3.3. Scoring Systems in PCC

Several PCC scoring systems have been proposed for CD patients undergoing SBCE, of which the Lewis score is the most widely used [10,20,21,22].

#### 3.3.1. Lewis Score (LS)

The LS metric has been used for the assessment of small bowel disease using SBCE and has been validated for use in clinical practice. Based on capsule transit time from the first duodenal image to the first caecal image, the small bowel is divided into three equal parts (tertiles). For each tertile, a subscore is determined based on the extent and distribution of oedema, as well as the number, size, and distribution of ulcers and the presence or absence of strictures. The LS is calculated from sum of the worst affected tertile plus the score of strictures, which are evaluated along the entire length of the small bowel, independently of the division in tertiles.

Software has been developed to enable readers to rapidly calculate the LS, and this ability is incorporated into the small bowel PillCam^®^ capsule RAPID READER^®^ (Version 8.3) reporting module. Cut-off values have been devised to grade and classify small bowel inflammatory activity: LS < 135 corresponds to a normal examination or non-significant inflammation; LS ≥ 135 and < 790 correspond to mild inflammation; and a LS ≥ 790 corresponds to moderate to severe activity [21].

#### 3.3.2. Pillcam Crohn’s Capsule Score

The Pillcam Crohn’s capsule score was developed by Eliakim to cover the lack of a quantitative activity index for the PillcamTM Crohn’s panenteric capsule [10]. The Panenteric Crohn’s capsule score (PCCS) was calculated using the reporting system embedded in the Rapid PillCam Reader software, version 9.0. The reporting system uses three tertiles for the small bowel (SB 1-3), left colon (LC), and right colon (RC) and measures the most common lesion (graded by severity as 1–3), the most severe lesion (graded by severity as 1–3), the approximated disease extent (none, 10–30%, 30–60%, 60–90%), and the presence of strictures (none, 1, >1, retention). There was a statistically significant correlation between panenteric Crohn’s capsule score and faecal calprotectin (r = 0.55, *p* < 0.001). Good interobserver agreement for the calculation of this score was shown, particularly for LS values > 135 (mild to severe inflammatory activity). For LS < 135, there was moderate agreement between readers (k = 0.58). For LS > 135 and LS > 350, the agreement was strong (k = 0.88 and 0.86, respectively, *p* < 0.001) [10].

#### 3.3.3. Capsule Endoscopy Crohn’s Disease Activity Index (CECDAI)

An additional score, known as the Capsule Endoscopy Crohn’s Disease Activity Index (CECDAI), has been proposed for the scoring of small bowel inflammation in CE. This score divides the small bowel into two segments based on the transit time of the capsule and includes the degree and extent of mucosal inflammation and the presence of strictures. The CECDAI evaluates three parameters: (a) inflammation (0–5-ulcers > 2 cm); (b) extent of disease (0–3-diffuse); and (c) strictures (0–3-obstruction). The final score is calculated by adding the two segmental scores: proximal ([A1 × B1] + C1) + distal ([A2 × B2] + C2). CECDAI < 3.8 corresponds to mucosal healing (LS < 135), while CECDAI > 5.8 corresponds to moderate-to-severe inflammation (LS ≥ 790). The correlation of these endoscopy indices with serum or faecal inflammatory markers has been shown to be poor. Faecal calprotectin has been shown to correlate slightly better with the LS compared to the CECDAI score (LS), especially for faecal calprotectin levels less than 100 µg/g, while at higher levels, there is little or no correlation between calprotectin levels and the LS. This finding suggests that patients may benefit from capsule endoscopy to assess disease extent and activity when their faecal calprotectin is greater than 100 µg/g [20,21].

#### 3.3.4. CECDALic Score

The CECDALic score was first described in 2018 and was able to measure the inflammatory activity in the small bowel and colon simultaneously. For the calculation of CECDAIic, the midpoint of the small bowel and colon is estimated utilising the transit time (small bowel transit time and colonic transit time) and dividing the small bowel into proximal and distal segments, as well as the colon in right and left segments. For each segment, capsule readers evaluate three parameters: (a) inflammation (0–5 ulcers > 2 cm); (b) extent of disease (0–3, diffuse); and (c) strictures (0–3, obstruction). The total score is achieved by multiplying the inflammation score (a) by the extent of disease score (b) and adding the stricture score (c) for each segment and finally by totalling them. A strong correlation between the CECDAIic score and calprotectin (r = 0.82; *p* = 0.012) and a moderate correlation with CRP (r = 0.50; *p* = 0.019) have been shown [22].

## 4. Discussion

Crohn’s disease is a patchy inflammatory disease that can affect any part of the gastrointestinal tract. In terms of distribution, 25% of patients have colitis only, 25% have ileitis only, and 50% have both small and large bowel involvement. With regard to small intestinal CD, SBCE is established as the most sensitive mucosal imaging modality when compared to barium follow-up, MRI, and CT enterography and colonoscopy with terminal ileoscopy. Unlike SBCE and ileo-colonoscopy, PCC delivers video images of the entire digestive tract, offering the opportunity to stage and monitor the disease from stomach to rectum with a single minimally invasive investigation [1,2,3]. This review indicates that, currently, there is considerable heterogeneity in the published reports. Studies are both single or multi-centre, using a variety of bowel cleansing, prokinetic, and boosting regimens and scoring systems. There is no uniform assessment of bowel cleansing, nor is there any indication of the experience of those reading the video reports [19] (Table 2).

Despite these inadequacies, the studies provide a useful overview of the potential of PCC to offer a single diagnostic investigation to grade the severity and distribution of inflammatory disease. The Tontini study suggested that the dual camera PCC increases diagnostic yield over single-camera SBCE, and the Eliakim PCCS score, which was designed to grade PCC rather than SBCE, provides a new benchmark for reporting on severity and distribution but requires further validation in other centres [8,10]. The large study reported by Bruining on PCC revealed greater sensitivity than ileo-colonoscopy and MR enterography and indicated a patient preference for PCC [15]. The Tai study demonstrated that PCC resulted in upstaging of grading and in just over one in three patients, resulting in a change of management [16]. The Olivia study in paediatric Crohn’s patients again demonstrated the potential value of PCC to grade and monitor disease activity and to alter treatment strategies in children undergoing biologic treatment [18,23].

PCC offers several advantages over traditional endoscopic and radiological procedures used to monitor CD [6]. Scanning the mucosal landscape of the small and large intestines in a single pass and the procedure do not require day-case hospital admission, intravenous access for sedation and analgesia, or pulse oximetry and post-procedure monitoring [1]. Unlike traditional endoscopy, little technical skill is required to deliver the device, while the expertise resides in reading and reporting the video images. There is concern about capsule retention, but with careful pre-assessment, the risk of capsule retention can be minimised by excluding patients with known severe stricturing disease or subacute obstructive symptoms. When there is uncertainty, pre-assessment with a patency capsule can forewarn of this possible complication. Intestinal obstruction occurring with capsule retention is rare, and most retained capsules pass spontaneously with intensification of treatment, but if there is concern, deep enteroscopy or surgical intervention is required [1]. The PCC provides an overview of disease activity and distribution, and in Adler’s study, interobserver agreement in >95.7% of UC patients was achieved regarding Mayo score [14]. However, the need for biopsy confirmation of diagnosis places the investigation downstream of the monitoring progress and, in particular, response to treatment.

The application of machine learning and artificial intelligence to videos generated by PCE has the potential to detect pathology with high accuracy and efficiency and substantially reduce reading times. Conventional reading of a complete small bowel video can take from 30 to 90 min, while AI algorithms can consistently reduce reading times to less than 30 min and, in some cases, even less than 10 min [24]. Furthermore, AI can reduce the risk of error due to factors such as bias, fatigue, or inexperience, and it can also improve training and learning opportunities by providing clinicians with only abnormal CE images for review [24]. Majter et al. showed that a deep learning framework in PCC was both sensitive and specific in identifying ulcers with high accuracy in both the small bowel and colon. Ulcerations were diagnosed with sensitivity, specificity, and diagnostic accuracy of 95.7% (CI 93.4–97.4), 99.8% (CI 99.2–100), and 98.4% (CI 97.6–99.0), respectively. The diagnostic accuracy was 98.5% (CI 97.5–99.2) for the small bowel and 98.1% (CI 96.3–99.2) for the colon. Ulcerations of different severities were classified with substantial agreement (κ = 0.72) [11].

## 5. Conclusions

The panenteric Crohn’s capsule was introduced in 2017. The limited published literature suggests that this minimally invasive procedure produces small and large bowel mucosal imaging with excellent sensitivity and specificity. While SBCE has cemented for itself a role in the monitoring of small bowel disease, the two-camera PCC extends capsule endoscopy reach into the colon. This reach offers Crohn’s patients requiring endoscopic staging and monitoring a gentler healthcare experience and the opportunity for clinicians to modify treatment. Challenges include the training of PCC readers, standardisation of preparation and booster regimens, further validation of scoring systems, and reducing time-consuming reading times. It is likely that, soon, the application of artificial intelligence to video image analysis will address reading time, as well as improve diagnostic accuracy and standardise assessment of bowel preparation and scoring. Is PCC ready for general use? The evidence suggests that, where appropriate, the answer is a considered “yes”.

## Figures and Tables

**Table 1:** Studies of the use of Pillcam Crohn’s Capsule in CD.

Author (Year)	Study Population	Study Design	Topic	Findings
Leighton et al., 2017 [12]	114	Prospective, multicentre	Comparison of diagnostic yield of PCE vs. IC for active CD lesions.	The diagnostic yield rate for active CD lesions was 83.3% for PCE and 69.7% for IC. PCE detection rates for active CD in the TI, caecum, ascending colon, transverse colon, descending/sigmoid colon, and rectum were 70, 38, 36, 30, 39, and 29%. IC was less sensitive with detection rates of 54, 26, 34, 24, 31, and 25%, respectively.
Eliakim et al., 2018 [13]	41	Prospective, multicentre	Feasibility study with a primary endpoint of successful video creation and report creation. The secondary endpoints were entire bowel inspection, duration of reading time, video quality, and adverse events.	All 41 videos met the primary endpoint. There was no capsule retention. Bowel coverage was graded 6.7 ± 0.6 and 6.1 ± 1.3 (1–7, unconfident–confident), image quality was graded 6.1 ± 0.8 (1–7, poor–excellent), and reading time was graded 3.7 ± 1.4 (1–7, very short to very long).
Adler et al., 2019 [14]	30	Prospective, multicentre	Comparison of PCE and colonoscopy in UC.	Moderate agreement for disease extent in UC in 56.5% of cases (kappa coefficient 0.42) with very good agreement for assessment of UC disease activity based on Mayo endoscopic subscore in 95.7% of cases (kappa coefficient 0.86).
Bruining et al., 2020 [15]	158	Prospective, multicentre	This study assessed the accuracy and safety of PCE in CD compared with IC and/or MRE.	PCE was equally sensitive to MRE and/or IC for active enteric inflammation (94 vs. 100%, *p* = 0.125) and more specific (74 vs. 22%, *p* = 0.001). The sensitivity of PCE was superior to that of MRE for enteric inflammation in the proximal small bowel (97 vs. 71%, *p* = 0.021) and similar to that of MRE and/or IC in the TI and colon.
Tontini et al., 2020 [8]	41	Prospective, multicentre	Comparison of diagnostic performance of PCC (with two cameras offering a 344° panoramic view) vs. the standard 172° view (one camera) in suspected or known CD.	PCC study completion rate of 90%. Compared with the standard 172°-view capsule, the panoramic 344°-view capsule showed that more patients had a relevant lesion (56.1% vs. 39.0%; *p* = 0.023), resulting in higher Lewis scores (222.8 vs. 185.7; *p* = 0.031), improving their clinical management (48.8% vs. 31.7%, *p* = 0.023)
Eliakim et al., 2020 [10]	41	Prospective, single-centre	The study evaluated the development of a novel Pillcam Crohn’s capsule score for the quantification of inflammation in the small bowel and colon in patients with CD	There was a high interrater reliability coefficient between the two readers for Lewis inflammatory and PillCam^TM^ Crohn’s score (0.9, *p* < 0.0001 for both). The correlation between PillCam^TM^ Crohn’s score and faecal calprotectin was stronger than for Lewis score (r = 0.32 and 0.54, respectively, *p* = 0.001 for both).
Majter et al., 2021 [11]	38	Prospective, multicentre	Detection and classification of CD using PCE using a deep learning framework	Deep learning approaches in PCE led to identification of ulcers with sensitivity of 95.4% and specificity of 98.4%. The diagnostic accuracy was 98.5% for the small bowel and 98.1% for the colon
Tai et al., 2021 [16]	93	Multicentre, observational study	PCC was used to evaluate the extent and severity of CD. The feasibility, safety, and impact on patient outcomes were also examined.	In 85% of cases, the examination was complete, and the PCC resulted in change of clinical management in 38.7% of patients. The Montreal classification was upstaged in 33.8% of patients with established CD, and mucosal healing was demonstrated in 15.5%. In 12.7% of patients, PCC upstaged the small bowel disease and predicted escalation of treatment.
Volkers et al., 2022 [17]	22	Prospective, multicentre	PCE was used to measure changes in mucosal disease activity before and after (8–12 weeks) starting biologic treatment in CD patients.	Endoscopic remission (absence of ulcers) was observed in 6 of 22 (27%) patients. 3 of 22 patients (59%) responded endoscopically, (50% decrease in SES-CD and CDEIS scores compared to baseline). No adverse effects were observed.
Oliva et al., 2020 [18]		Prospective, multicentre	PCC was used to evaluate the extent and severity of paediatric IBD population.	At baseline, active inflammation was seen in 34 patients (71%), in 22 patients (46%) at week 24, and in 18 patients (39%) at week 52. PCC led to treatment change in 34 patients (71%) at baseline and 11 patients (23%) at 24 weeks.

**Table 2:** PCE bowel cleansing regimens.

Author (Year)	Bowel Prep	Dietary Instructions	Prokinetic Used	Boosters Used
Leigthton et al., 2017 [12]	4 L PEG	Clear liquid diet for 24 h	Metoclopramide 10 mg (optional)	Suprep+ 2 L of water+ bisacodyl
Eliakim et al., 2018 [13]	4 L PEG	Clear liquid diet for 24 h	Metoclopramide 10 mg (optional)	Suprep/Picosalax + bisacodyl
Adler et al., 2019 [14]	3 L PEG	Low residue diet for 12 h, fasting for 12 h	Metoclopramide 10 mg (optional)	Half bottle (88 mL) of sodium sulphate, potassium sulphate, and magnesium sulphate solution
Bruining et al., 2020 [15]	4 L PEG	Clear liquid diet for 24 h	Metoclopramide 10 mg or erythromycin 250 mg (optional)	Suprep
Tontini et al., 2020 [8]	2 L PEG	Clear liquid diet for 24 h, fasting for 12 h	None	None
Eliakim et al., 2020 [10]	4 L PEG	Clear liquid diet for 24 h	Metoclopramide 10 mg (optional)	Suprep/Picosalax + bisacodyl
Majter et al., 2021 [11]	4 L PEG	Overnight fasting	None	None
Tai et al., 2021 [16]	NA	NA	NA	NA
Volkers et al., 2022 [17]	3 L PEG (split dose) + 10 mg bisacodyl 2 days before the procedure	Low-fibre diet 2 days prior to test	None	Phosphoral (sodium phosphate) or picoprep (sodium picosulphate and magnesium citrate) with 1–2 L of clear fluid afterwards

## Data Availability

Not applicable.
